# Euscaphic Acid from *Rosa roxburghii* Tratt Fruit Ameliorates Ulcerative Colitis by Modulating Oxidative Stress, Inflammation, Gut Microbiota, and Metabolism

**DOI:** 10.3390/foods15203730

**Published:** 2026-10-20

**Authors:** Jinlong Liu, Qi Wang, Xuefeng Bian, Yu Han, Jingyao Liang, Rongxin Zhang, Yu Xiao, Quanquan Chen, Xiaomei Zhang, Xin Sun

**Affiliations:** School of Pharmaceutical Sciences, Jilin Medical University, No. 5, Jilin Street, Fengman District, Jilin 132013, China; jinlongliu@jlmu.edu.cn (J.L.); wangqicvb@163.com (Q.W.); shaole2024@163.com (X.B.); hanyu.jlmu@vip.163.com (Y.H.); 18843416985@163.com (J.L.); 15567887261@163.com (R.Z.); xiaoyu20051111@163.com (Y.X.); 18897776875@163.com (Q.C.)

**Keywords:** euscaphic acid, *Bacteroides uniformis*, ulcerative colitis, multi-omics

## Abstract

Although euscaphic acid (EA)—the main active component of *Rosa roxburghii* Tratt fruit—possesses potential antioxidant, anti-inflammatory, and intestinal protective effects, the mechanisms underlying these properties remain unclear. This study aimed to investigate whether EA can alleviate dextran sulfate sodium (DSS)-induced ulcerative colitis (UC) through multiple mechanisms such as inhibiting oxidative stress, regulating inflammation, remodeling the gut microbiota, and restoring metabolic disturbances. Using a DSS-induced mouse model of UC and integrating histopathological examination, Western blotting, 16S rRNA sequencing, untargeted metabolomics, transcriptome sequencing, and fecal microbiota transplantation, EA’s mechanisms of action were investigated at multiple levels, including the tissue, microbial, and metabolic levels. EA counteracted DSS-induced alterations in MPO, SOD, MDA, IL-6, IL-1β, and TNF-α. In addition, EA intervention improved UC and reversed the reduction in *Bacteroides uniformis* (*B. uniformis*) in UC mice. Further studies confirmed that *B. uniformis* is a key species mediating the UC-alleviating effects of EA. Metabolomics analysis indicated that EA could mitigate the effects of metabolic disorders; transcriptomic analysis indicated that it inhibits inflammatory signaling pathways; and multi-omics analysis further revealed that it alleviates UC by restoring the gut microbiota and correcting metabolic disorders while simultaneously inhibiting oxidative and pro-inflammatory pathways.

## 1. Introduction

Inflammatory bowel disease, which includes Crohn’s disease and ulcerative colitis (UC), has a relatively high incidence and mortality rate in European and American countries [1]. Due to the inflammatory nature of UC, if it is not treated appropriately and promptly, it can lead to persistent intestinal damage and increase the risks of surgery and colorectal cancer [2]. Research has shown that alterations in the gut microbiota are closely associated with UC [3]. When the microbial balance is disrupted, bacteriocins produced by pathogenic bacteria can damage intestinal barrier function; furthermore, abnormalities in the composition and function of proteins such as Occludins and the Zonula Occludens (ZO) family lead to compromised epithelial barrier integrity and trigger inflammation [4,5].

Presently available drug treatments for UC, such as 5-aminosalicylic acid and glucocorticoids, can cause adverse reactions [6]. In addition to conventional drug therapy, probiotics and fecal microbiota transplantation (FMT) from healthy donors have emerged as promising alternatives for treating UC [7,8]. However, their high costs pose significant challenges to broader application. People are increasingly concerned not only with the basic nutrients needed for human growth but also with the health and therapeutic functions of nutritional foods, such as antioxidant, anti-cancer, and anti-inflammatory properties [9]. Diet has become a key regulator of disease onset and progression, with functional nutritional food components potentially influencing intestinal inflammation, barrier function, and microbial composition [10,11]. The assessment of underutilized functional nutritional foods is an important strategy for maintaining human dietary nutrition and addressing the challenges associated with UC.

*Rosa roxburghii* Tratt fruit (RRTF) is the mature fruit of *Rosa roxburghii*, a species of the genus Rosa in the family Rosaceae [12,13,14]. This plant is a shrub whose fruit is oblate-spherical, about 3–4 cm in diameter; when mature, the fruit is yellow-green and its peel is densely spiny, with erect persistent sepals [15,16]. The functional nutritional components of RRTF mainly include polysaccharides, superoxide dismutase, flavonoids, triterpenes, and amino acids [17,18], which have antioxidant, anti-inflammatory, and antitumor effects [9,19,20]. Its decoction can be used to treat chronic gastritis, stomach pain, acute enteritis, diarrhea, and dysentery in both humans and animals [21]. Euscaphic acid (EA) is one of the most abundant triterpenoid acids found in the RRTF, exhibiting anti-inflammatory activity. Recent studies have shown that EA protects vascular endothelial cells, alleviates skin inflammation, relieves itching, enhances antioxidant activity, and mitigates fatigue and inflammation, demonstrating significant therapeutic effects for these conditions [22,23,24]. However, its efficacy and mechanisms in UC have not yet been fully studied.

In this study, we systematically evaluated the effects of EA on UC using a multi-omics approach. Overall, the results indicate that the ameliorative effect of EA on UC is associated with a multidimensional network involving the reduction in oxidative stress, inhibition of inflammation, and restoration of the gut microbiota and metabolic disturbances, providing a natural candidate therapeutic for UC with the advantage of a pleiotropic mechanism. In summary, our research findings indicate that EA may serve as a potential therapeutic agent for UC treatment, providing additional insights into the underutilized functional nutritional food RRTF.

## 2. Materials and Methods

### 2.1. Experimental Reagents

EA was extracted and isolated from RRTF in our laboratory (the HPLC fingerprint of EA is in Figure S1, with a purity > 95%). An EA standard product was purchased from Chengdu DeSiTe Biological Technology Co., Ltd. (53155-25-2, Chengdu, Sichuan, China). Sulfasalazine (SASP) was purchased from Solarbio Biotechnology Ltd. (S3940, Beijing, China). The dextran sulfate sodium (DSS) was purchased from MP Biomedicals (9011-18-1, Solon, OH, USA). *Bacteroides uniformis* (*B. uniformis*) was purchased from the China General Microbiological Culture Collection Center (CGMCC 1.5133, Beijing, China). Interleukin-6 (IL-6), Interleukin-1β (IL-1β), and Tumor Necrosis Factor-alpha (TNF-α) kits were purchased from Beyotime Biotechnology (PI326, PI301, PT512, Shanghai, China). Myeloperoxidase (MPO), Malondialdehyde (MDA), and Superoxide Dismutase (SOD) kits were purchased from Nanjing Jiancheng Bioengineering Institute (H508-1-2, A003-1-2, A001-3-2, Nanjing, China). Details of all antibodies used are provided in Supplementary Table S1.

### 2.2. In Vivo Experimental Design

Seven-week-old male C57BL/6J mice were purchased from QIANHE BIOTECH Co., Ltd (Changchun, China). All animal experiments in this study were approved by the Animal Ethics Committee of Jilin Medical University (Permit No. 2024-GKJJ-019, 11 March 2024). Humane endpoints and euthanasia criteria, including body weight loss exceeding 20%, were established in advance, and observations, weighing, and record-keeping were performed by trained personnel. Mice were housed in a sterile environment at a temperature of 21–23 °C and a humidity of 50–55%, with adequate sterile feed and purified water provided, under a 12-h light/12-h dark cycle. The methods for establishing the DSS-induced UC mouse model, gut microbiota depletion, and FMT were based on the previously published literature [25].

**Figure 1:**
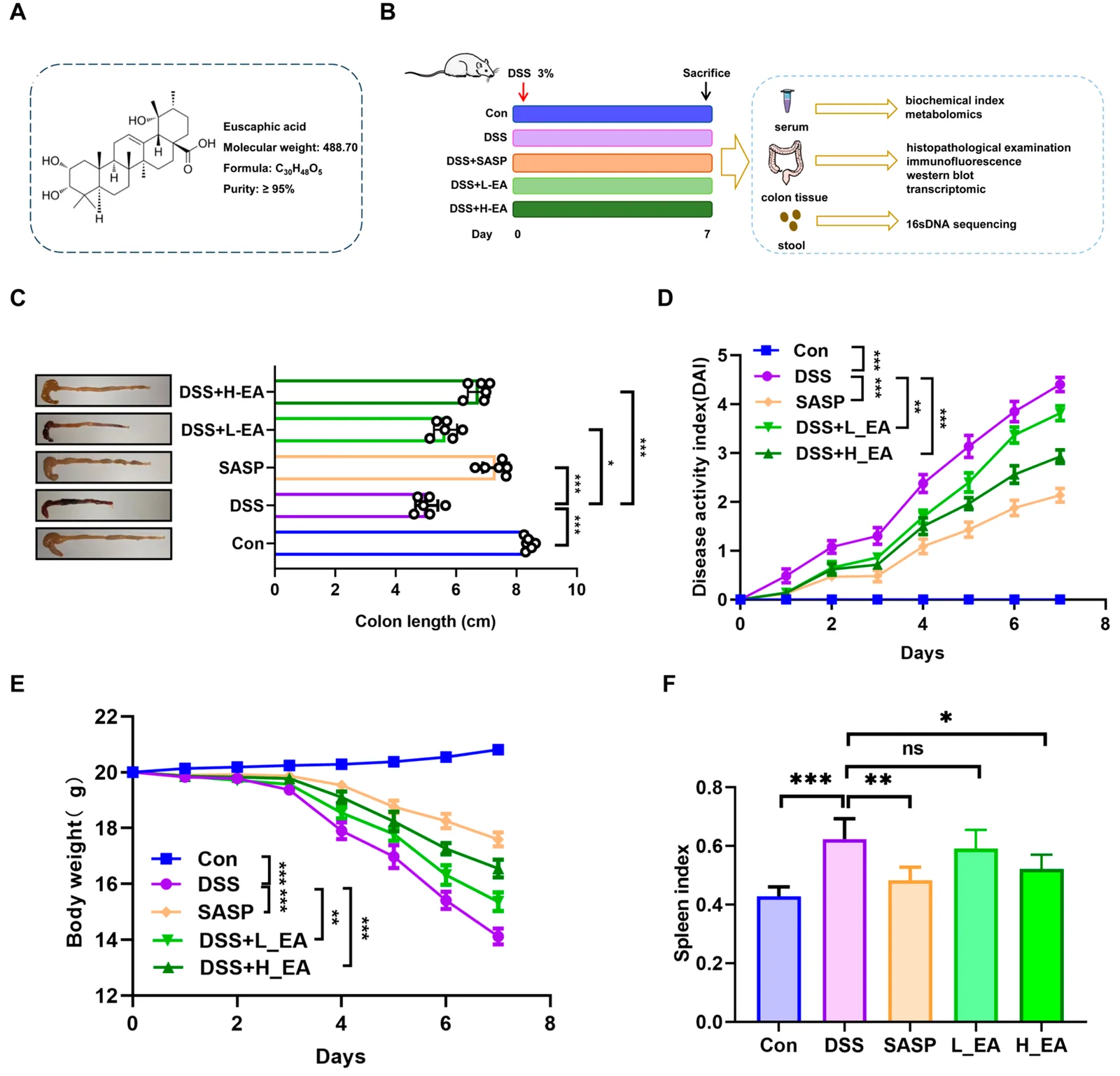
EA ameliorates UC symptoms. (**A**) Chemical structure of EA. (**B**) Experimental design plan. (**C**) Intuitive diagram of colon. (**D**) Disease activity index (DAI) scores. (**E**) Weight change. (**F**) Spleen index. Data are expressed as mean ± SD (*n* = 6 for each group). * *p* < 0.05, ** *p* < 0.01, *** *p* < 0.001. ns: non-significance.

All mice were acclimated for 7 days with adequate access to purified water and food. Figure 1 shows the chemical structure of EA and the detailed experimental procedure for EA intervention in animals. The mice were randomly divided into five groups—normal control group, model control group (DSS, 3%), positive control group (SASP 250 mg/kg), low-dose treatment group (EA 10 mg/kg), and high-dose treatment group (EA 20 mg/kg)—with 6 mice per group according to a random number table method. Except for the control group, all other groups freely drank a 3% DSS aqueous solution, while the treatment groups received daily intragastric administration. Following administration of 3% DSS, mice were monitored daily for general condition, activity level, food and water intake, stool characteristics, hematochezia, dehydration, and changes in body weight. The time of death or attainment of humane endpoints was recorded. During the modeling period, mice exhibited progressive weight loss. When body weight loss exceeded 20% of the pre-administration weight on day 7, the predefined humane endpoint was considered reached, and euthanasia was performed on day 7. To alleviate animal suffering, mice were monitored daily, provided with readily accessible food and water, and maintained in an appropriate and quiet housing environment. Euthanasia was performed immediately, with no further experimental observation, if mice showed marked weakness, severe dehydration, persistent hypoactivity, inability to eat or drink, or body weight loss meeting the endpoint criterion. After 7 consecutive days of administration, feces were collected for 16S rRNA gene analysis, and blood samples were obtained to isolate sera for biochemical and metabolomic analyses. Meanwhile, colon tissues were collected for histopathological examination and transcriptome sequencing.

### 2.3. Gut Microbiota Depletion and EA Supplementation Experiment

Mice were randomly divided into three groups—a model control group (DSS, 3%), an antibiotics (ABX) group, and an ABX + EA (20 mg/kg) group—with 6 mice per group according to a random number table method. To deplete the gut microbiota, in some groups, drinking water was supplemented with an antibiotic cocktail (0.2 g/L of ampicillin, neomycin, and metronidazole and 0.1 g/L of vancomycin) for the whole duration of the experiment [26]. After 7 days of gavage, blood was collected for serum biochemical analysis and colon tissues were subjected to histopathological examination.

### 2.4. FMT Experiment

The mice were assigned randomly to three groups—(1) DSS (3%) group; (2) DSS (3%) + FMT group; and (3) DSS (3%) + EA_FMT group—with 6 mice per group according to a random number table method. Feces (600 mg) from EA mice were mixed with 6 mL of sterile saline, and the supernatant was obtained by centrifugation at 1000× *g*. Mice were treated with ABX via oral gavage for 7 days to deplete the gut microbiota [26], followed by oral gavage of feces from EA donors for 6 days [27]. Subsequently, sera and colons were collected.

### 2.5. B. uniformis Intervention Experiment

Mice were randomly divided into three groups—a model control group (DSS, 3%), *B. uniformis* (10^8^ CFU) group, and *B. uniformis* (10^8^ CFU) + EA (20 mg/kg) group—with 6 mice per group according to a random number table method. After 7 days of gavage, blood was collected for serum biochemical analysis, and colon tissues were subjected to histopathological examination.

### 2.6. Immunohistochemistry

The prepared sections were incubated in a 60 °C oven for 1 h to rewarm. They were then dewaxed in xylene and rehydrated through a graded ethanol series, with each step lasting 2 min. Antigen retrieval was performed by heating the sections in sodium citrate buffer (pH 6.0) to a boil in a microwave oven for 20 min. Endogenous peroxidase activity was subsequently blocked with 3% hydrogen peroxide. The sections were then subjected sequentially to blocking, primary antibody incubation, secondary antibody incubation, and 3,3′-diaminobenzidine chromogenic development. Nuclear counterstaining was performed with hematoxylin. Following rinsing with deionized water, the sections were dehydrated with 80%, 95%, and absolute ethanol, cleared in xylene, and mounted with neutral resin for microscopic examination.

### 2.7. Statistical Analysis

Values are expressed as the mean ± standard deviation (SD) from three or six independent experiments. One-way analysis of variance (ANOVA) was used to compare three or more groups. n denotes the number of biological replicates; specifically, *n* = 6 for in vivo measurements in mice and sequencing. In the figures, significance is indicated as * *p* < 0.05, ** *p* < 0.01, *** *p* < 0.001.

Additional methods are provided in the Supplementary Material.

## 3. Results

### 3.1. Therapeutic Effects of EA on UC

To investigate the ameliorative effect of EA (Figure 1A) on the progression of UC, we administered EA to DSS-treated mice via oral gavage (Figure 1B). During DSS-induced UC, EA supplementation markedly delayed colon shortening, alleviated diarrhea and rectal bleeding, and significantly reduced weight loss (Figure 1C–E). In addition, EA suppressed the increase in spleen index (Figure 1F). These data suggest that EA treatment alleviates the symptoms of UC.

### 3.2. EA Ameliorates DSS-Induced Inflammation and Oxidative Stress and Improves Intestinal Barrier Function

**Figure 2:**
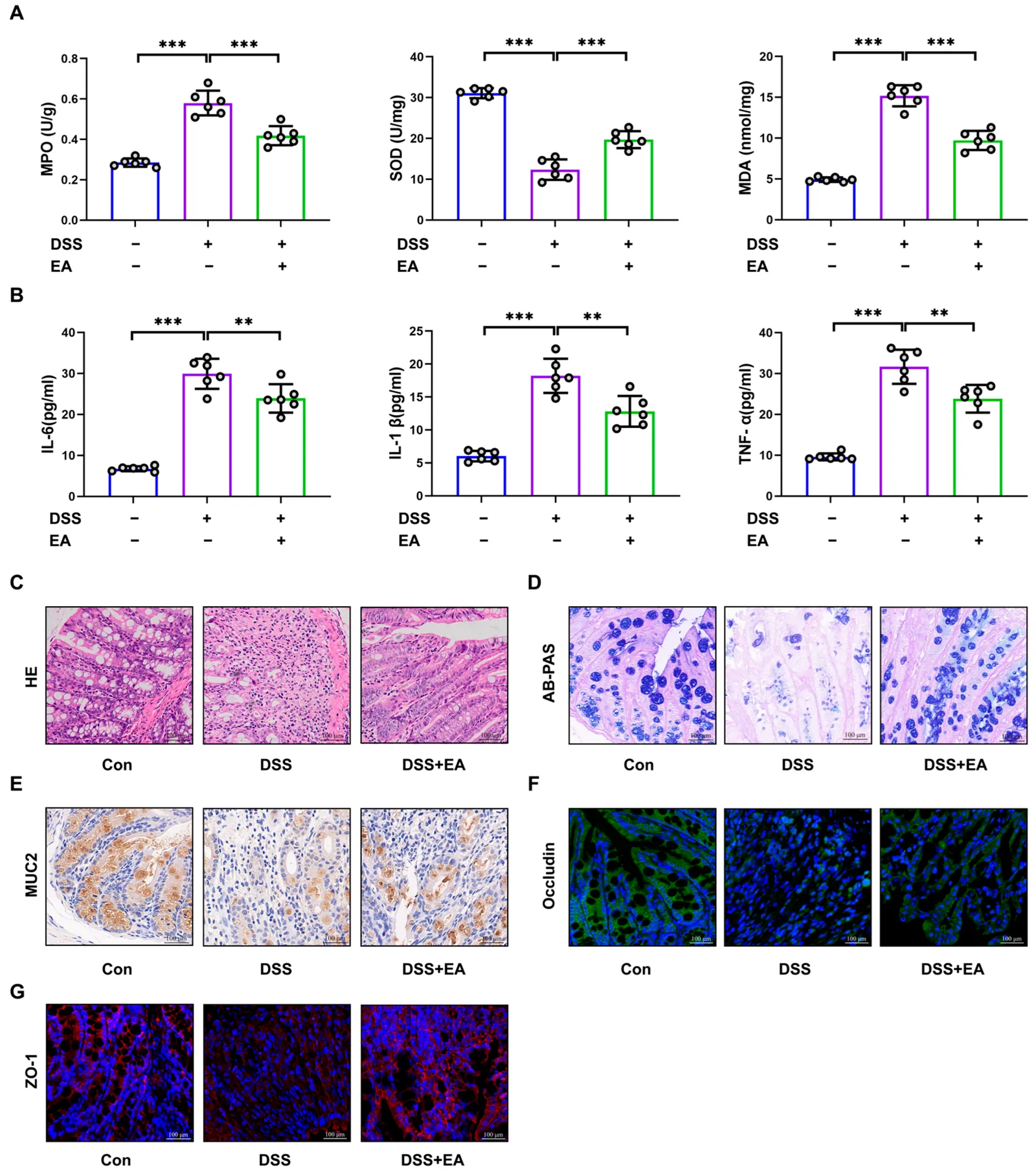
Ameliorative effects of EA on oxidative stress, inflammation, and intestinal barrier function in UC mice. (**A**) Colon MDA, SOD, and MPO contents. (**B**) Serum inflammatory factors and IL-6, IL-1β, and TNF-α levels. (**C**) Colon H&E staining (400×). (**D**) Representative AB/PAS-stained sections (400×). (**E**) Representative MUC2-stained sections (400×). (**F**,**G**) Representative Occludin and ZO-1-stained sections (400×). Data are expressed as mean ± SD (*n* = 6 for each group). ** *p* < 0.01, *** *p* < 0.001.

It is well known that DSS can increase oxidative stress and lead to excessive production of inflammatory factors. We found that EA can enhance SOD expression in colonic tissues and reduce the levels of MPO and MDA (Figure 2A). In addition, Figure 2B shows that EA intervention can suppress the levels of inflammatory factors. These results clearly indicate that EA alleviates DSS-induced inflammation and oxidative stress injury.

Impaired intestinal barrier function is considered an important factor in UC. We found that DSS stimulation caused significant damage to the mouse colon, with a severe reduction in the number of goblet cells and infiltration of inflammatory cells. Histological analysis further showed that pathological damage in the mouse colon was markedly alleviated following EA treatment (Figure 2C). EA also reversed the reduction in goblet cell number and restored the expression of mucin 2 (MUC2), Occludin, and ZO-1 (Figure 2D–G). Additionally, EA significantly reduced serum levels of LPS and FITC-dextran (Figure S2).

### 3.3. EA Reverses Gut Dysbiosis in UC

**Figure 3:**
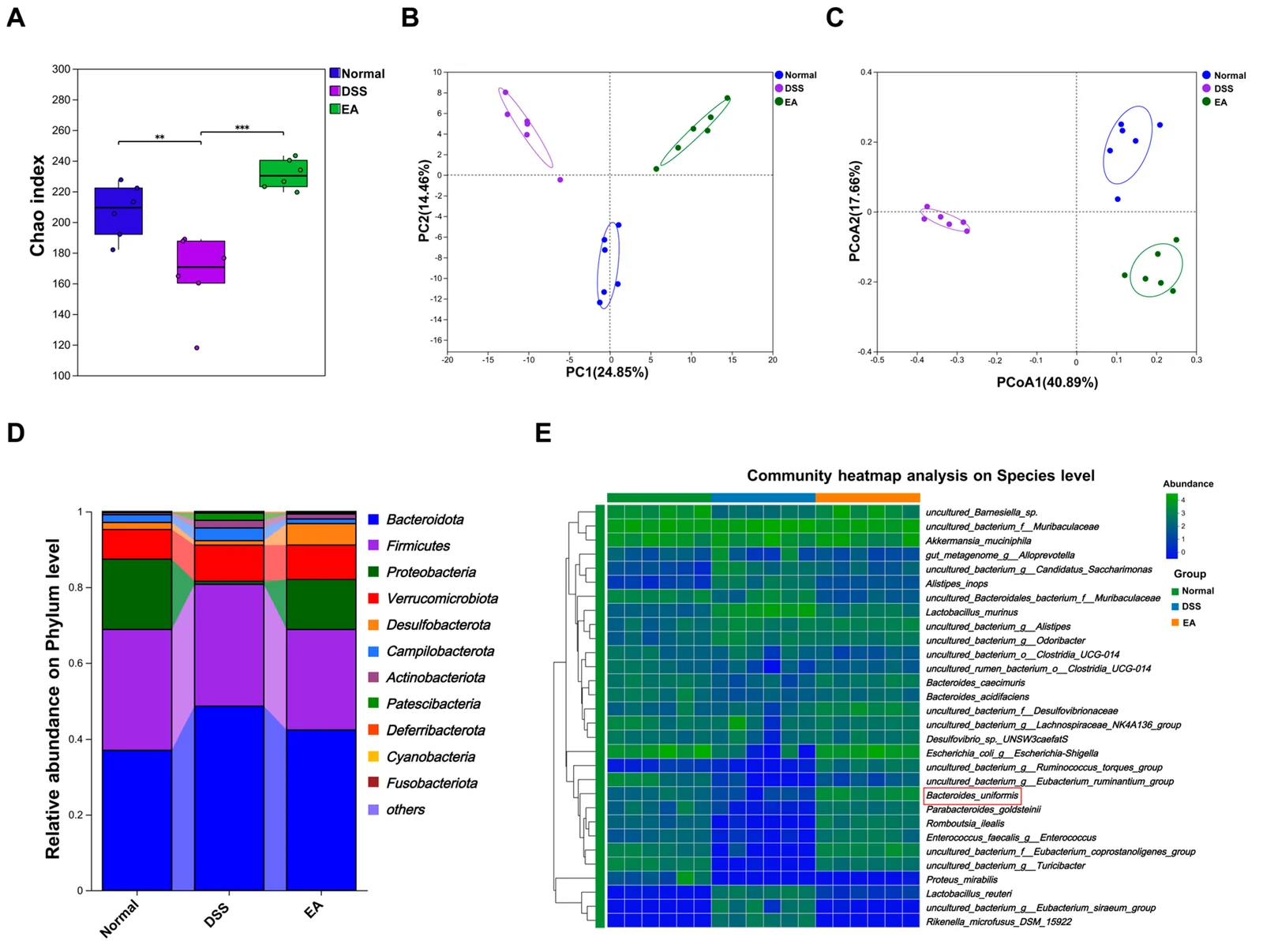
EA regulates the intestinal microbiota composition. (**A**) Chao indices. (**B**,**C**) Principal component analysis (PCA) and Principal coordinate analysis (PCoA) analyses. (**D**) Phylum level of microorganisms. (**E**) Community heatmap analysis at species level. ** *p* < 0.01 and *** *p* < 0.001.

To assess the effects of EA treatment on the gut microbiome, we performed 16S rRNA gene sequencing. Alpha and beta diversity analyses showed that EA restored community richness and diversity, with separation among the groups (Figure S3A and Figure 3A–C). At the phylum level, DSS increased the relative abundance of *Bacteroidota* and decreased *Proteobacteria* and *Desulfobacterota*, whereas EA reversed these changes (Figure 3D). At the genus level, *Lactobacillus* abundance increased and *Escherichia-Shigella* abundance decreased in the model group, while EA reversed these trends (Figure S3B). At the species level, the abundance of *B. uniformis* decreased in the model group, and EA reversed this change (Figure 3E). In summary, EA restored the gut microbiota’s balance and increased beneficial bacteria.

### 3.4. EA Alleviates UC, Which Is Related to Gut Microbiota

**Figure 4:**
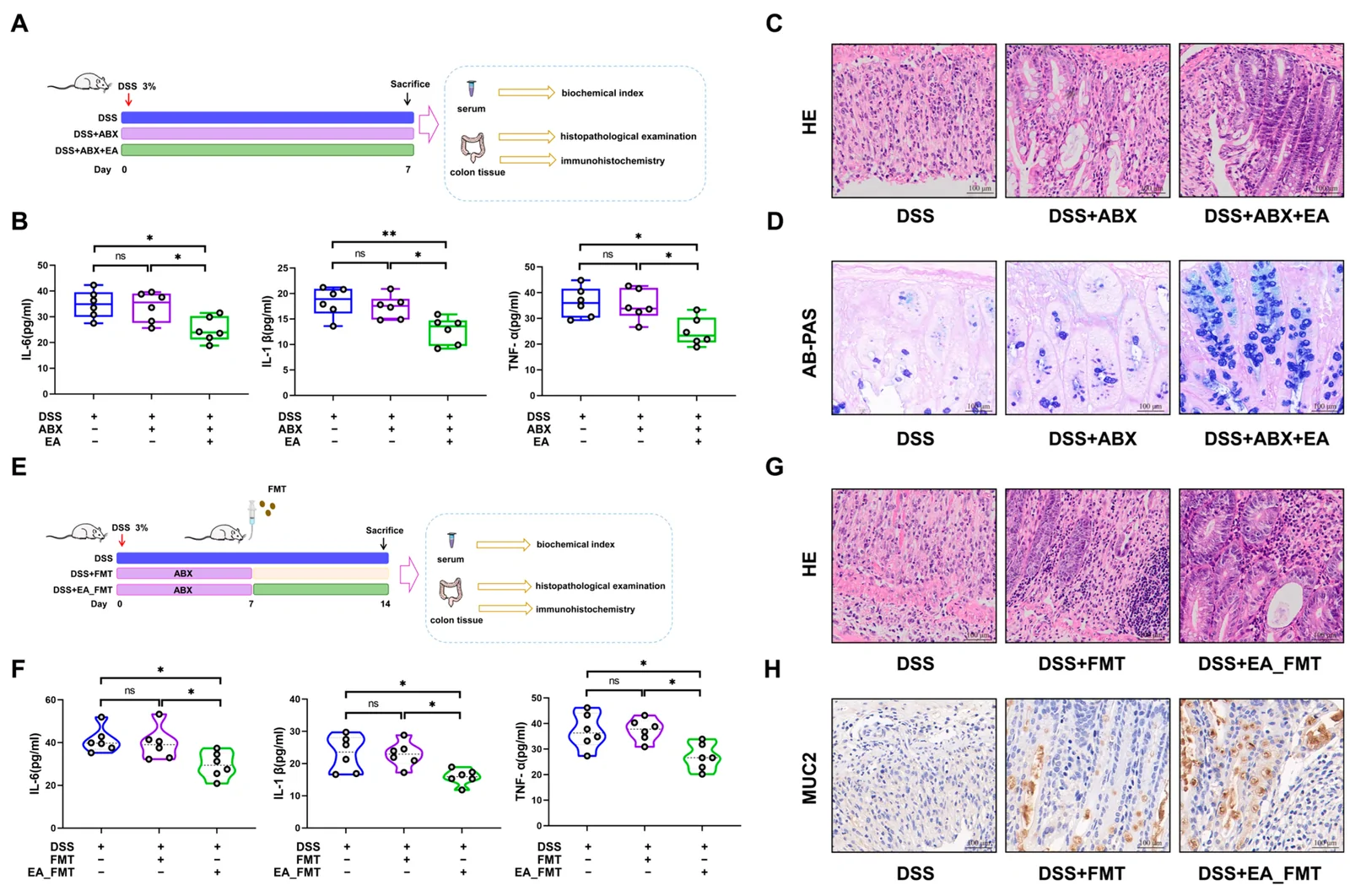
Influence of the EA-modulated gut microbiota on UC. (**A**) Experimental schema for germ-free mice. (**B**) Serum inflammatory factor levels. (**C**) Colon H&E staining (400×). (**D**) Representative AB/PAS-stained sections (400×). (**E**) Experimental design for FMT. (**F**) Serum inflammatory factor levels. (**G**) Colon H&E staining. (**H**) Representative MUC2-stained sections (400×). * *p* < 0.05, ** *p* < 0.01. ns: non-significance.

To examine whether gut microbiota remodeling contributes to the protective effect of EA, we first depleted the intestinal bacteria via ABX treatment (Figure 4A) and successfully established a microbiota-depleted mouse model (Figure S4). Compared with the DSS-induced model mice without ABX intervention, ABX-treated model mice showed no differences in inflammatory factor levels, inflammatory infiltration, or the number of colonic goblet cells (Figure 4B–D). With ABX intervention, EA treatment further improved colonic injury severity (Figure 4B–D). FMT experiments were subsequently performed (Figure 4E). Crucially, EA_FMT alleviated inflammation and protected against intestinal barrier dysfunction (Figure 4F–H). These data indicate that EA ameliorates UC in part by correcting the gut microbiota dysbiosis, although its protective effects were not entirely microbiota-dependent.

### 3.5. B. uniformis Is a Key Functional Bacterium Associated with EA Treatment

**Figure 5:**
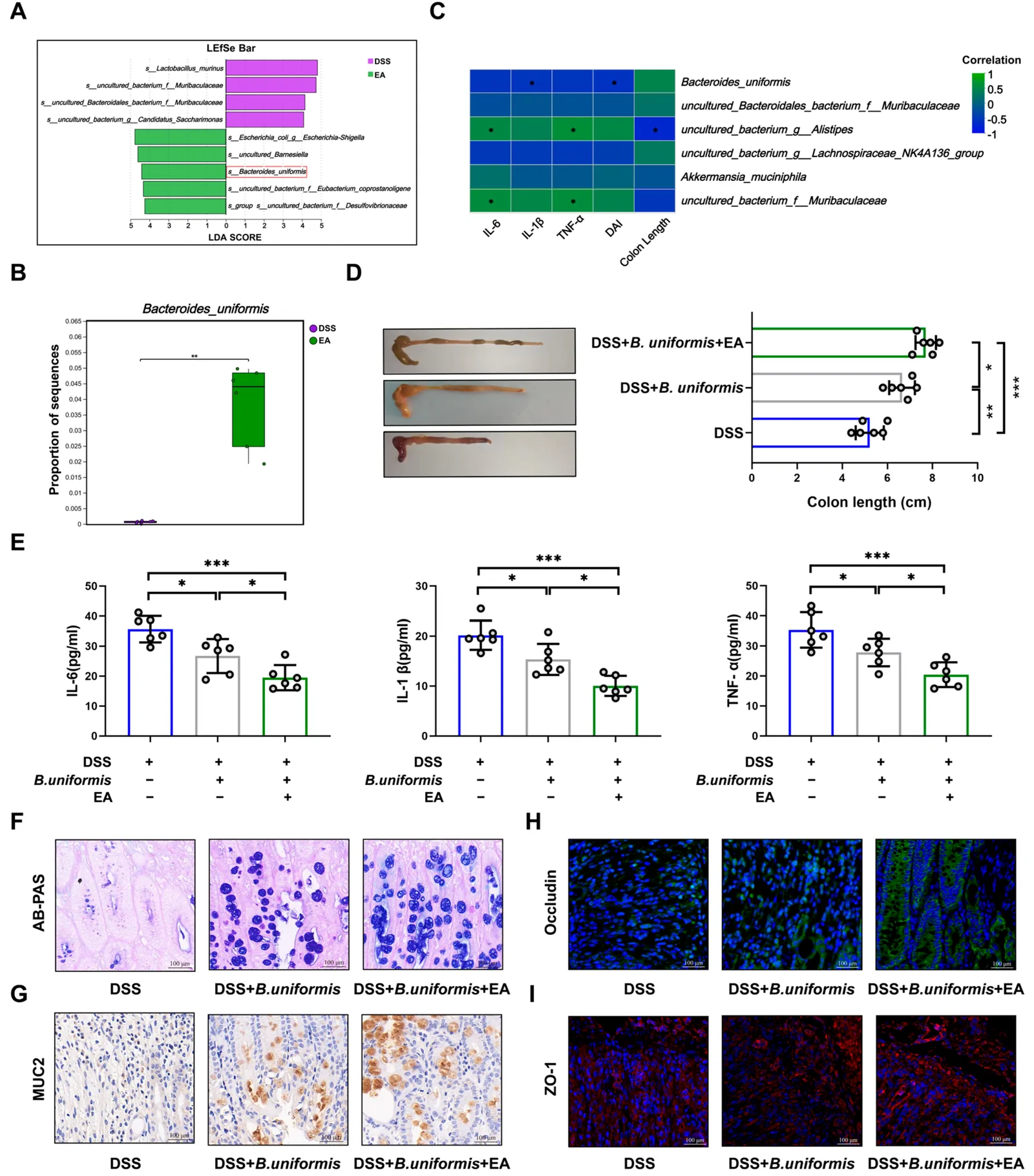
EA promoted *B. uniformis*, jointly improving UC. (**A**) LEfSe analysis. (**B**) Abundance of *B. uniformis*. (**C**) Heatmap of the correlation between bacteria and UC-related parameters. (**D**) Intuitive diagram of colon. (**E**) Serum inflammatory factor levels. (**F**) Representative AB/PAS-stained sections (400×). (**G**) Representative MUC2-stained sections (400×). (**H**,**I**) Representative Occludin and ZO-1-stained sections (400×). * *p* < 0.05, ** *p* < 0.01, *** *p* < 0.001.

Next, we examined the key bacterial species involved in EA-mediated UC alleviation at the species level. EA treatment significantly modulated the bacterial community (Figure S5). In mice treated with EA, the abundance of *B. uniformis* increased significantly, and this species was identified as a microbial feature of the EA group (Figure 5A,B). Spearman rank correlation analysis further showed that *B. uniformis* was positively correlated with colon length and negatively correlated with the levels of IL-6, IL-1β, TNF-α, and DAI (Figure 5C). Increasing research suggests that *B. uniformis* has a beneficial effect on UC [28,29,30]. Based on these findings, we speculate that *B. uniformis* contributes to the improvement of UC by EA. Further in vivo studies found that *B. uniformis* alleviated colon shortening in UC mice, reduced inflammatory cytokine levels, and restored DSS-induced intestinal barrier dysfunction (Figure 5D–I). These results indicate that *B. uniformis* contributes to the improvement of UC by EA.

### 3.6. EA Remodels Metabolic Dysregulation in UC Mice

**Figure 6:**
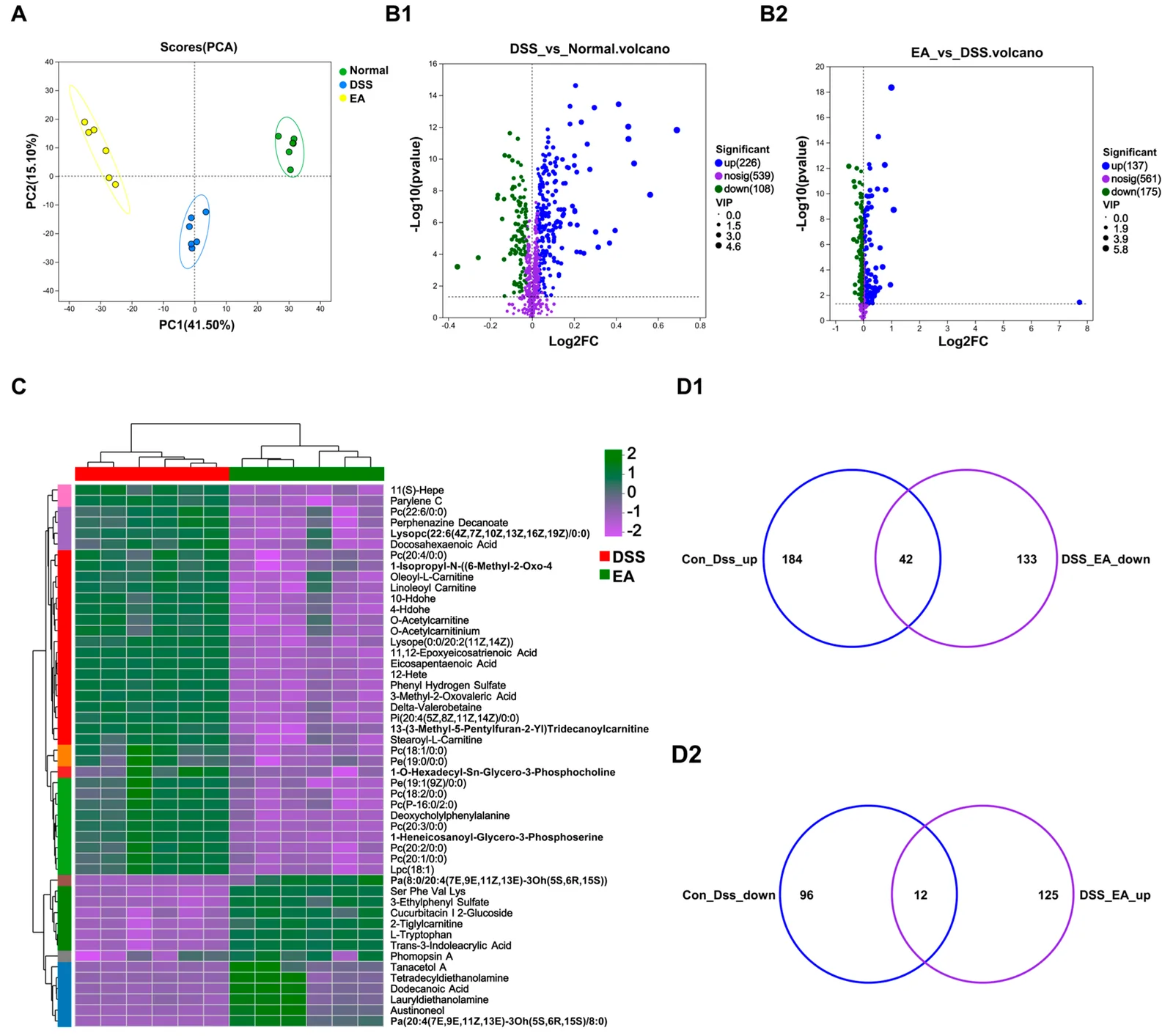
Non-targeted metabolomics indicates that EA intervention alters the metabolites in the serum. (**A**) PCA of metabolites. (**B1**,**B2**) Volcano map analysis. (**C**) Cluster heatmap of DAMs in DSS vs. EA. (**D1**,**D2**) Venn diagram of DAMs.

We performed untargeted metabolomics analysis to assess the metabolic profiles of different groups. A total of 665 annotated metabolites were identified based on Human Metabolome Database (HMDB), and PCA revealed clear separation among groups (Figure S6A and Figure 6A). Compared with control mice, DSS-induced colitis significantly altered metabolite levels, whereas EA markedly restored these metabolites (Figure 6(B1,B2)). Additionally, clustering heatmaps for the DSS vs. normal and EA vs. DSS group comparisons are shown in Figure S6B and Figure 6C.

To clarify the metabolic basis of EA-mediated protection against DSS-induced UC, we further analyzed differentially abundant metabolites (DAMs). Figure 6(D1,D2) show that there were 42 DAMs that were increased after DSS treatment and subsequently decreased by EA, and 12 DAMs that were decreased after DSS treatment and subsequently increased by EA. This indicates that EA can reverse the metabolic disturbances associated with UC.

### 3.7. EA Regulates Transcriptional Levels and Inhibits Inflammatory Signaling Pathways in UC

**Figure 7:**
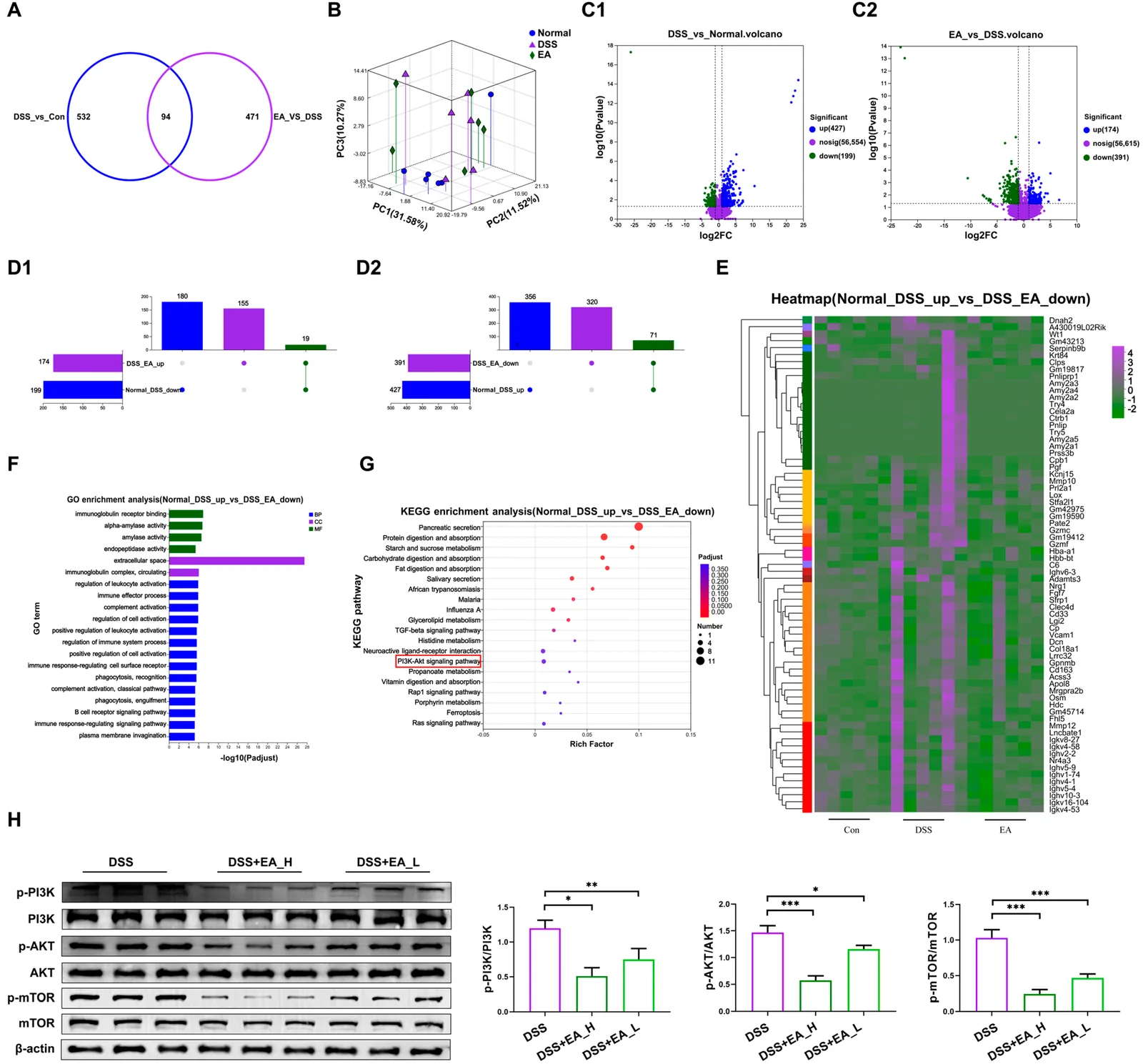
EA inhibits the expression of colon oxidative stress and pro-inflammatory pathways. (**A**) Venn diagram of DEGs. (**B**) PCA. (**C1**,**C2**) Volcano plot of DEGs. (**D1**,**D2**) Upset plot of DEGs. (**E**) Clustering thermal map of significant DEGs activated by DSS but inhibited by EA. (**F**) GO functional annotation analysis of DEGs activated by DSS but inhibited by EA. (**G**) KEGG enrichment analysis of DEGs activated by DSS but inhibited by EA. (**H**) Western blot analysis of p-PI3K, p-AKT, p-mTOR, PI3K, AKT, mTOR, and β-actin protein expression. * *p* < 0.05, ** *p* < 0.01, *** *p* < 0.001.

To investigate the possible mechanisms underlying the protective effects of EA on DSS-induced UC, we performed transcriptomic analysis. The Venn diagram shows that 94 differentially expressed genes (DEGs) were shared between the two transcriptomes (Figure 7A). PCA demonstrated that the transcriptional profiles of each group differ significantly (Figure 7B). After DSS treatment, 427 DEGs were up-regulated and 199 DEGs were down-regulated. After EA intervention, 174 DEGs were up-regulated and 391 DEGs were down-regulated (Figure 7(C1,C2)). The clustering analysis in Figure S7A,B shows significant differences between the groups. As shown in Figure 7(D1), DSS suppressed the expression of 19 genes, whereas EA enhanced their expression. Conversely, after DSS treatment, the expression of 71 genes increased, but after EA intervention, the expression of these genes decreased (Figure 7(D2)). We observed clear cluster separation among the groups (Figure S7C and Figure 7E). The DEGs down-regulated by EA treatment are mainly associated with categories such as “immunoglobulin receptor binding,” “extracellular space,” and “regulation of leukocyte activation” (Figure 7F). KEGG analysis identified the PI3K-AKT signaling pathway as an EA-enriched pathway (Figure 7G). Importantly, EA treatment inhibited the expression of p-PI3K, p-AKT, and p-mTOR (Figure 7H).

## 4. Discussion

RRTF is a natural plant fruit unique to Guizhou Province in China that is edible and nutritionally rich. Recently, researchers have conducted an in vitro simulation of oral digestion to analyze the release and overall bioavailability of antioxidant components [31]. We conducted more in-depth in vivo studies on the effects of EA—a major active component of the natural plant RRTF, possessing strong antioxidant and anti-inflammatory properties—in ameliorating UC and its underlying mechanisms. We found that EA, by modulating the gut microbiota and metabolic dysregulation, significantly reduced oxidative stress and inflammation and promoted the restoration of intestinal barrier function, thereby effectively suppressing UC. Importantly, the dose of EA that produced the above improvements was non-toxic and caused no side effects (Figure S8).

UC is accompanied by recurrent diarrhea, bloody purulent stools, and impaired intestinal barrier function. After EA treatment, reduced levels of LPS and FITC-dextran were observed in the serum. Both the EA and EA_FMT treatments increased the expression of barrier function-related proteins, highlighting their beneficial effects on intestinal barrier function. Notably, the EA treatment increased the abundance of the beneficial bacterium *B. uniformis* in the UC model. An increasing number of studies indicate that *B. uniformis* has anti-inflammatory effects [28,29]. This suggests that EA improves UC by enhancing *B. uniformis*. However, in germ-free mice, EA further suppressed inflammation and intestinal barrier dysfunction. These data indicate that the beneficial effects of EA on UC are driven by both microbiota-dependent and -independent mechanisms.

Given that UC occurs alongside metabolic disorders, we conducted a non-targeted metabolomics analysis, the results of which indicated that EA restored metabolic imbalances. We found that DSS caused a reduction in tryptophan, while tryptophan levels significantly increased following EA treatment. Tryptophan is an essential amino acid, which is directly converted by gut microorganisms into various molecules, such as indole and its derivatives, in order to maintain the stability of the intestinal environment [32]. This highlights the impact of DSS administration and the regulatory effects of EA treatment [33]. Given EA’s ability to restore metabolic dysregulation in UC, we further investigated the signaling mechanisms underlying EA’s UC-ameliorating effects through expression analysis. RNA sequencing analysis showed that EA inhibited PI3K-Akt signaling pathways in UC. The PI3K-Akt signaling pathway is widely considered a central hub connecting upstream inflammatory factors and downstream cell fate, with an increasing number of studies demonstrating its crucial role in UC [34,35,36]. These results clearly demonstrate that EA modulates microbiota imbalance and metabolic disturbances and suppresses inflammation, thereby improving UC.

Given the difficulty of achieving remission and the notable adverse effects of existing therapies, there is an urgent need to develop UC treatments that are both safe and effective. Compared with conventional UC therapies, EA demonstrates moderate regulatory effects, multiple therapeutic effects, and no side effects. Although our study demonstrated that EA modulated the microbiota, further research is needed to determine the effects of metabolites of *B. uniformis* on UC and their clinical applications. This is crucial for fully understanding the anti-inflammatory mechanisms of EA and for advancing its development as a therapeutic agent.

## 5. Conclusions

In summary, we demonstrated for the first time that an active component of RRTF—namely, EA—alleviates the effects of UC in an in vivo model. EA modulates the composition of the gut microbiota, restores metabolic homeostasis, ameliorates the effects of UC, and suppresses pro-inflammatory pathways. Our study provides guidance for the therapeutic application of the active components of RRTF in improving UC. The development and utilization of triterpene compounds from RRTF is expected to be of great significance for the comprehensive utilization of RRTF, providing insights for the development of functional nutritional foods in the future.

## Data Availability

The original contributions presented in the study are included in the article and Supplementary Materials, further inquiries can be directed to the corresponding authors. The raw 16S rRNA gene and RNA-seq data are freely available in the NCBI database under accession no. PRJNA1280100 and PRJNA1247716.

## Supplementary Materials

The following supporting information can be downloaded at: https://www.mdpi.com/article/10.3390/foods15203730/s1, Table S1: Antibodies used in this study; Figure S1: HPLC fingerprint of EA; Figure S2: EA restores DSS-induced gut barrier dysfunction. ((**A**) and (**B**)) Serum FITC-dextran 4 KD concentration and LPS concentration of control and EA treated mice from DSS model; Figure S3: EA regulates intestinal microbiota composition. (**A**) Venn diagram; (**B**) Genus level of microorganisms; Figure S4: Alpha diversity of gut microbiota in DSS treated mice after Abx treatment; Figure S5: Kruskal–Wallis test; Figure S6: Cluster heatmap of DAMs. (**A**) HMDB database classification; (**B**) cluster heatmap of DAMs in DSS vs. normal; Figure S7: Clustering thermal map of DEGs. (**A**) show that clustering thermal map of significant DEGs of DSS vs. Normal; (**B**) show that clus-tering thermal map of significant DEGs of DSS+ EA vs. DSS; (**C**) show that clustering thermal map of significant DEGs inhibited by DSS but activated by EA; Figure S8: Pathological effects of high-dose EA treatment on the liver, kidney, and spleen of mice.
