# Supplementary material for: Euscaphic Acid from Rosa roxburghii Tratt Fruit Ameliorates Ulcerative Colitis by Modulating Oxidative Stress, Inflammation, Gut Microbiota, and Metabolism
Source: Foods. 2026 Oct 20;15(20):3730. doi: 10.3390/foods15203730 (PMC13704157; doi:10.3390/foods15203730)
Supplement: Supplementary file 1 [file foods-15-03730-s001.zip › foods-4558466-supplementary.pdf]

Euscaphic Acid from Rosa roxburghii Tratt Fruit Ameliorates Ulcerative Colitis by Modulating Oxidative Stress, Inflammation, Gut Microbiota, and Metabolism

Jinlong Liu †, Qi Wang †, Xuefeng Bian, Yu Han, Jingyao Liang, Rongxin Zhang, Yu Xiao, Quanquan Chen, Xiaomei Zhang * and Xin Sun *

School of Pharmaceutical Sciences, Jilin Medical University, No. 5, Jilin Street, Fengman District, Jilin 132013, China; jinlongliu@jlmu.edu.cn (J.L.); wangqicvb@163.com (Q.W.); shaole2024@163.com (X.B.); hanyu.jlmu@vip.163.com (Y.H.); 18843416985@163.com (J.L.); 15567887261@163.com (R.Z.); xiaoyu20051111@163.com (Y.X.); 18897776875@163.com (Q.C.)

* Correspondence: 101012@jlmu.cn (X.Z.); sunxin@jlmu.edu.cn (X.S.)

† These authors contributed equally to this work.

Supplementary methods

Supplementary figure legends:

Table S1. Antibodies used in this study.

Figure S1. HPLC fingerprint of EA.

Figure S2. EA restores DSS-induced gut barrier dysfunction. ((**A**) and (**B**)) Serum FITC-dextran 4 KD concentration and LPS concentration of control and EA treated mice from DSS model.

Figure S3. EA regulates intestinal microbiota composition. (**A**) Venn diagram; (**B**) Genus level of microorganisms.

Figure S4. Alpha diversity of gut microbiota in DSS treated mice after Abx treatment.

Figure S5. Kruskal-Wallis test.

Figure S6. Cluster heatmap of DAMs. (**A**) HMDB database classification; (**B**) cluster heatmap of DAMs in DSS vs. normal.

Figure S7. Clustering thermal map of DEGs. (**A**) show that clustering thermal map of significant DEGs of DSS vs. Normal. (**B**) show that clustering thermal map of significant DEGs of DSS+ EA vs. DSS. (**C**) show that clustering thermal map of significant DEGs inhibited by DSS but activated by EA.

Figure S8. Pathological effects of high-dose EA treatment on the liver, kidney, and spleen of mice.

Supplementary Methods

Biochemical Assays

All biochemical tests were conducted in accordance with the instructions, and the OD values were measured using a microplate reader. IL-6, IL-1β, TNF-α, MPO, MDA, and SOD kits were purchased from Nanjing Jiancheng Bioengineering Institute (Nanjing, China) or Beyotime Biotechnology (Shanghai, China).

Spleen Index

Carefully dissect the connective tissue and fat surrounding the spleen with forceps to re move the spleen intact. Immediately place the removed spleen into pre-chilled PBS, rinse to remove blood, blot the surface moisture and saline solution from the spleen with filter paper, and weigh it on a precision electronic balance. Record the wet weight of the spleen. Spleen index (%) = (spleen weight / mouse body weight) x 100%.

EA Extraction and Insolation Procedures

Fresh *Rosa roxburghii* fruit (60 kg) was washed and chopped, then extracted under reflux with 95% ethanol three times for 2–3 h each. The extracts were combined and concentrated under reduced pressure until no alcohol odor remained. An appropriate amount of distilled water was added to disperse the residue, which was then extracted successively with petroleum ether, ethyl acetate, and n-butanol. The ethyl acetate extract was concentrated under reduced pressure to give 1,953 g of extract. The ethyl acetate fraction was subjected to gradient elution on a silica gel column with petroleum ether–acetone (50:1–0:1). Fractions with the same composition were combined based on TLC analysis to give seven fractions (A1–A7). Fraction A6 (petroleum ether–acetone, 3:1) was subjected to gradient elution on a silica gel column with petroleum ether–acetone (15:1–0:1), yielding four subfractions (A6.1–A6.4). Fraction A6.2 was eluted from a silica gel column with petroleum ether–acetone (7:1). TLC analysis was used to combine fractions with the same composition, yielding compound 3 (152 mg), which was identified as EA by comparison with an authentic standard. HPLC analysis showed that its purity was greater than 95% (Figure S1).

16S rRNA Gene Expression Analysis

In this study, gut microbiota analysis was performed on six intestinal content samples randomly selected from each experimental group. Genomic DNA was extracted from bacterial communities in the samples, followed by amplification of the V3–V4 hypervariable region of the 16S rRNA gene using specific primers. Paired-end sequencing was carried out on the Illumina MiSeq platform. Raw sequencing reads were assembled into longer contiguous sequences using FLASH v1.2.11, and quality filtering was performed using QIIME 1.9.1 to remove low-quality reads, chimeras, and artifacts. The sequencing depth of all samples was uniform, with an average of 48,016 effective sequences. High-quality sequences were clustered into operational taxonomic units at a 97 % sequence similarity threshold with UPARSE 7.0, and taxonomic annotation was assigned using the SILVA reference database. Downstream bioinformatics analyses, including alpha and beta diversity assessments, microbial composition profiling, and statistical comparisons, were conducted using the MajorBio Cloud Platform (www.majorbio.com), ensuring reproducibility and standardization of data processing.

Metabolomics Analysis

Serum samples were spiked with methanol-acetonitrile (1:1, v/v, containing internal standard L-2-chlorophenylalanine) for metabolite extraction, followed by centrifugation,

nitrogen blow-drying, reconstitution, and instrumental analysis. All sample supernatants were mixed in equal amounts to prepare QC quality control samples, which were used to monitor sample preparation and instrument stability. A UHPLC QExactiveHF X high-resolution mass spectrometry system was used to acquire ESI positive and negative ion signals, respectively. Raw data underwent peak identification, retention time correction, and peak alignment using ProgenesisQI v3.0. Metabolite annotation was performed using HMDB, METLIN, and an in-house mass spectral library, with primary ion mass error less than 10 ppm, and qualitative analysis completed by matching MS/MS secondary fragmentation spectra. Metabolites were annotated based on Level B (i) and B (ii) criteria. PCA and OPLS-DA multivariate statistical analyses were conducted, and differential metabolites were screened using VIP ≥ 1 and $P < 0.05$. KEGG pathway enrichment was performed using hypergeometric distribution. Data reliability was assessed by QC sample RSD and internal standard Z-score. Metabolomics analysis of samples was performed at Majorbio Biomedical Technology Co., Ltd. (Shanghai, China), following the manufacturer's protocols.

RNA Sequencing

Colon tissue samples were collected from each group of mice (n = 6 per group) and sent to Majorbio Bioinformatics Technology Co., Ltd, (Shanghai, China), for RNA sequencing analysis. Total RNA was extracted from each colon tissue sample using TRIzol Reagent (Life Technologies, Carlsbad, CA, USA) following the manufacturer's instructions. RNA quality and concentration were evaluated by examining RNA degradation and contamination on 1% agarose gels and measuring the 260/280 nm ratio using the NanoDrop 2000 spectrophotometer (IMPLEN, Calabasas, CA, USA). For transcriptome sequencing, 2 μg of RNA per colon tissue sample was used as the input material. The NEBNext Ultra RNA Library Prep Kit (NEB, Beverly, MA, USA) was used to generate sequencing libraries according to the manufacturer's guidelines. The libraries were sequenced on an Illumina HiSeq 2500 platform (Illumina, San Diego, CA, USA), generating paired-end reads with a length of 150 bp. Differential expression analysis was performed to identify genes with significant changes, using a cutoff criterion of $\log2|\text{fold-change}| > 1$ and ($P < 0.05$). Functional enrichment analysis on the annotated differentially expressed genes (DEGs) was carried out using software Cluster Profiler (v4.2.2). Additionally, by utilizing gene ontology (GO) pathway categories with a significance level ($P < 0.05$), genes with potential significance within the identified modules were obtained.

Histopathological Examination and Immunofluorescence

Colon tissues were fixed in 4% paraformaldehyde for 48 h, dehydrated, embedded in paraffin, and sectioned at 4 μm. The prepared sections were placed in a 60°C oven for 1 h, then deparaffinized in xylene and rehydrated through a graded ethanol series, 2 min at each step. The sections were subsequently stained with hematoxylin for 3 min, rinsed with deionized water, then stained with eosin for 5 min. After rinsing with deionized water, the sections were dehydrated through 80%–95%–absolute ethanol, cleared in xylene, and finally mounted with neutral resin for microscopic examination. Sections were first incubated with primary antibodies against Occludin and ZO-1 for 1 h, washed, and then incubated with the corresponding secondary antibody. After an additional wash, one drop of Gelvatol was added, and sections were examined after 30 min.

Alcian Blue-Periodic Acid SCHIFF Staining

Tissue sections were stained with Alcian blue (AB) staining solution (pH 2.5) for 5–10 minutes, rinsed with water, and then stained with PAS staining solution A (periodic acid) for 5 minutes, followed by rinsing with distilled water. The sections were

subsequently stained with PAS staining solution B (Schiff reagent) for 5–10 minutes and rinsed with distilled water. After dehydration, deparaffinization, and mounting, the sections were examined under a microscope.

Intestinal Permeability Detection

Intestinal permeability was evaluated using fluorescein isothiocyanate–dextran with a molecular weight of 4 kDa (FD4). Briefly, mice were fasted overnight before receiving FD4 by oral gavage at a dose of 500 mg/kg, prepared in PBS. Four hours after administration, approximately 100 μL of blood was collected via orbital sinus puncture. Serum fluorescence was measured using a microplate reader at an excitation wavelength of 485 nm and an emission wavelength of 535 nm. Serum FD4 concentrations were calculated from a standard curve generated using serially diluted FD4 solutions. Following euthanasia, serum lipopolysaccharide (LPS) concentrations were quantified using a mouse LPS ELISA kit.

Western Blotting

Colon tissue was lysed on ice, and protein samples were quantified. After electrophoresis and membrane transfer, the PVDF membrane is incubated with the target antibody, washed, and then incubated with the secondary antibody. Subsequently, the membrane was visualized and stored, and quantitative analysis was performed using ImageJ.

Table S1. Antibodies used in this study.

Antibodies	Source	Identifier
Anti- Phospho-PI3K	Cell Signaling Technology	Cat#4228
Anti- PI3K	Proteintech	Cat#27921-1-AP
Anti- Phospho-AKT	Proteintech	Cat#28731-1-AP
Anti- AKT	Proteintech	Cat#10176-2-AP
Anti- Phospho-mTOR	Proteintech	Cat#67778-1-IG
Anti- mTOR	Proteintech	Cat#28273-1-AP
Anti-MUC2	Abcam	Cat#ab97386
Anti-Occludin	Abcam	Cat#ab216327
Anti-ZO1	Proteintech	Cat#21773-1-AP
Anti-β-actin	Proteintech	Cat#66009-1-Ig

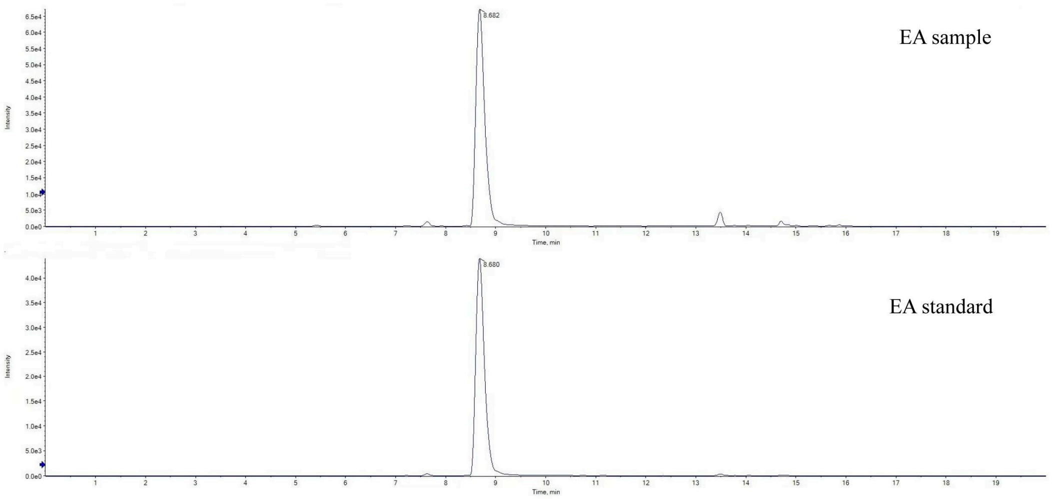

Figure S1. HPLC fingerprint of EA.

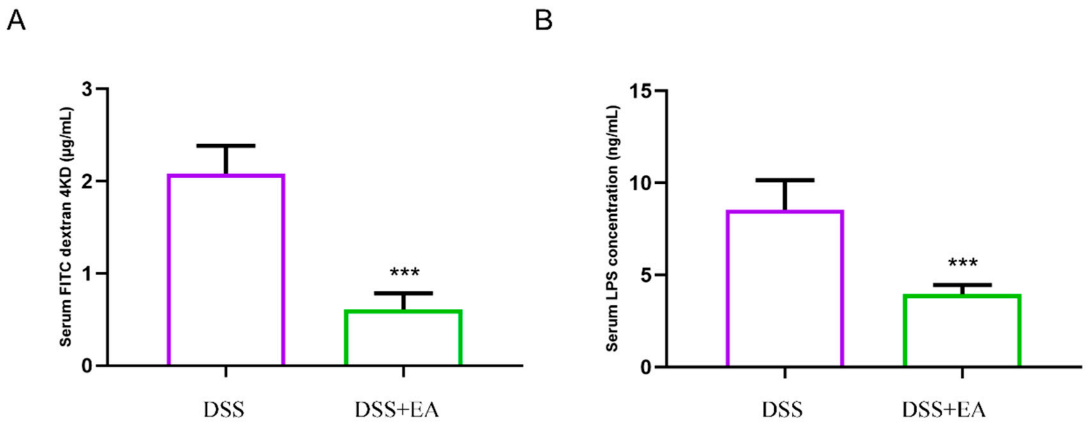

Figure S2. EA restores DSS-induced gut barrier dysfunction. ((**A**) and (**B**)) Serum FITC-dextran 4 KD concentration and LPS concentration of control and EA treated mice from DSS model.

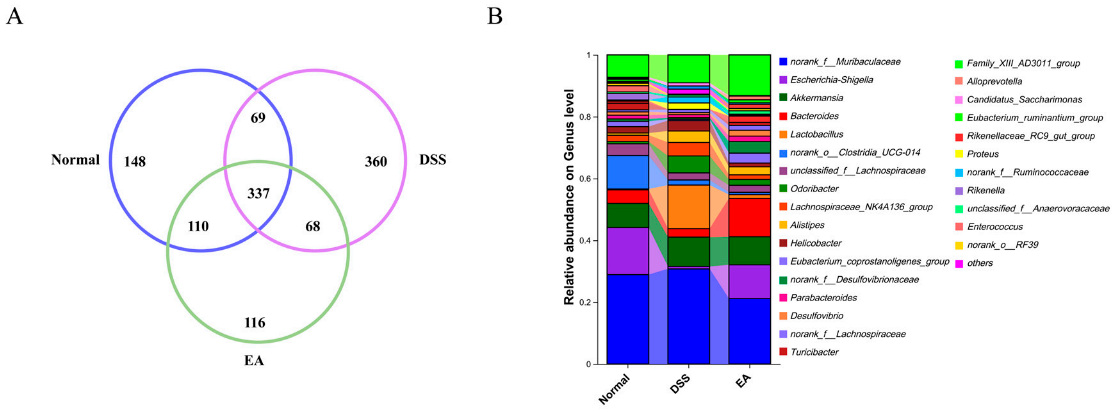

Figure S3. EA regulates intestinal microbiota composition. (**A**) Venn diagram; (**B**) Genus level of microorganisms.

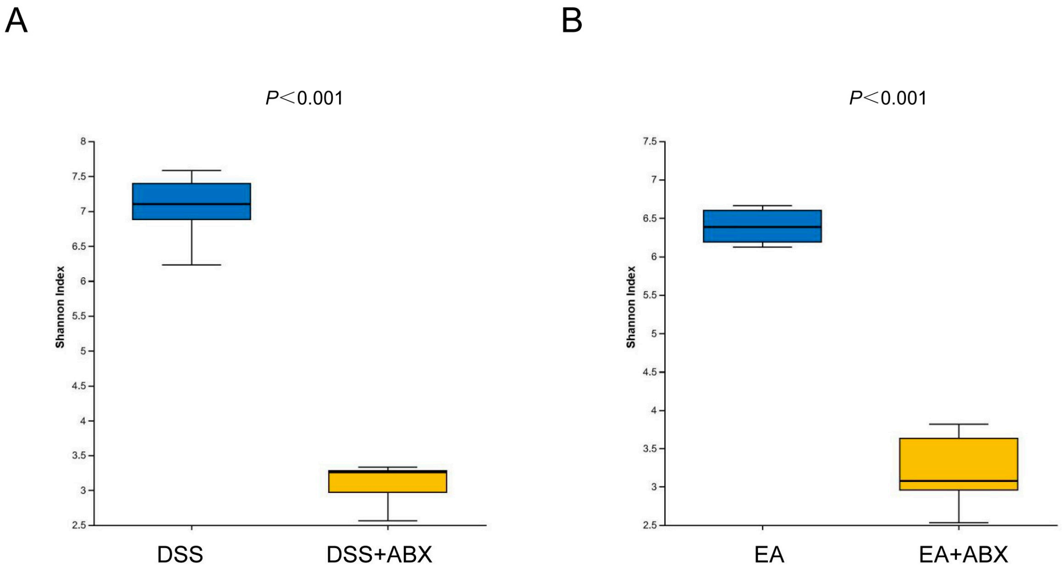

Figure S4. Alpha diversity of gut microbiota in DSS treated mice after Abx treatment.

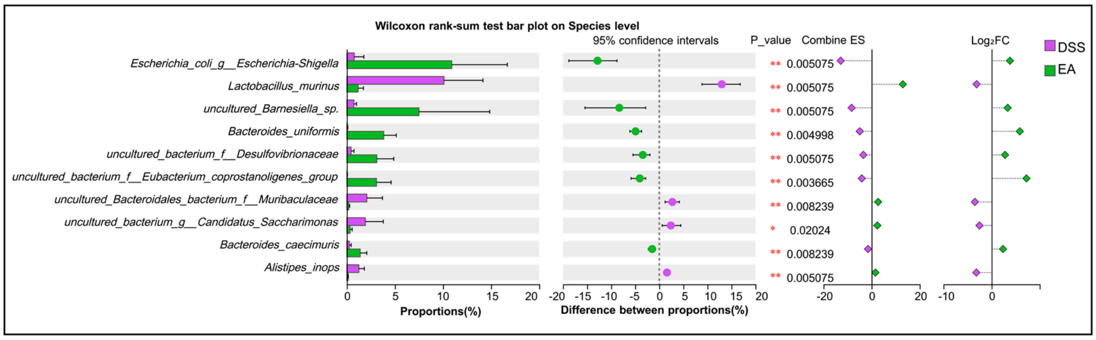

Figure S5. Kruskal-Wallis test.

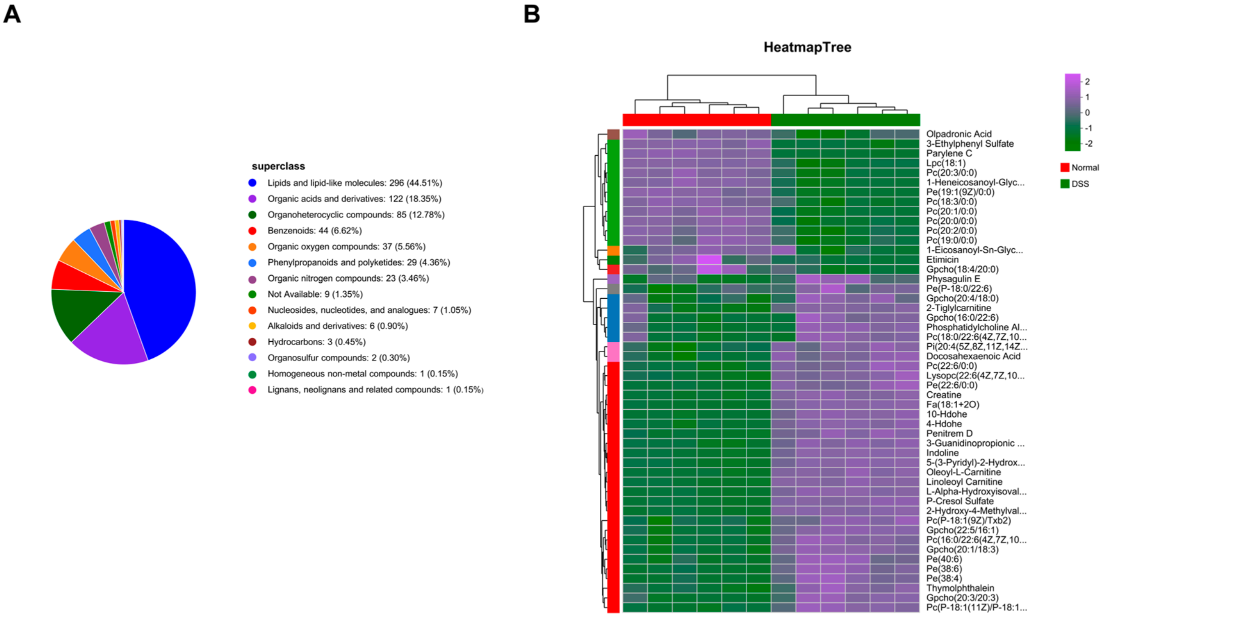

Figure S6. Cluster heatmap of DAMs. (**A**) HMDB database classification; (**B**) cluster heatmap of DAMs in DSS vs normal.

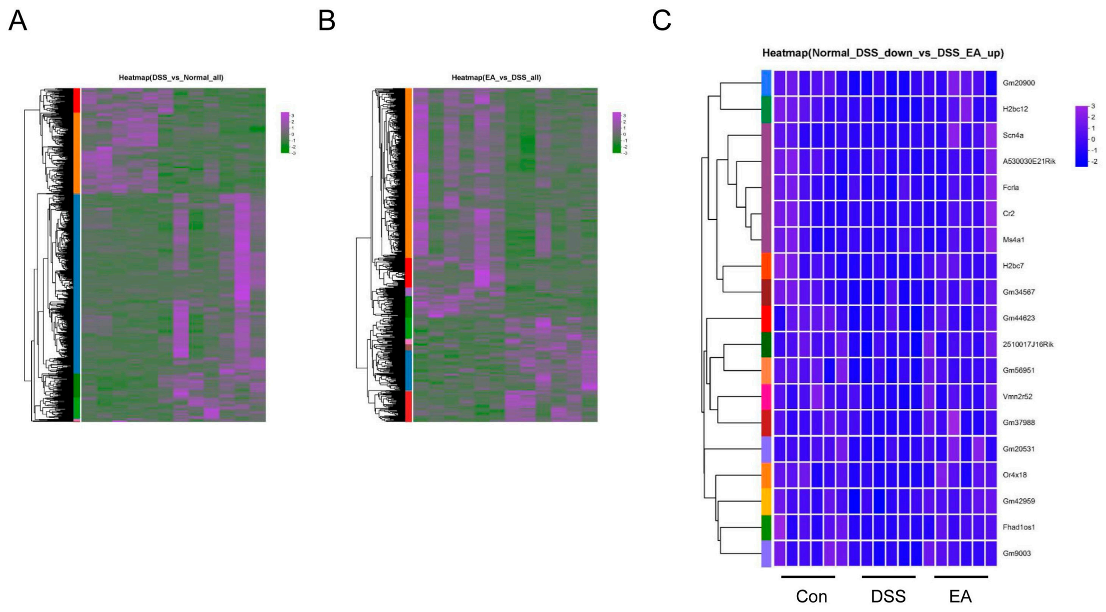

Figure S7. Clustering thermal map of DEGs. (**A**) show that clustering thermal map of significant DEGs of DSS vs Normal; (**B**) show that clustering thermal map of significant DEGs of DSS+ EA vs DSS; (**C**) show that clustering thermal map of significant DEGs inhibited by DSS but activated by EA.

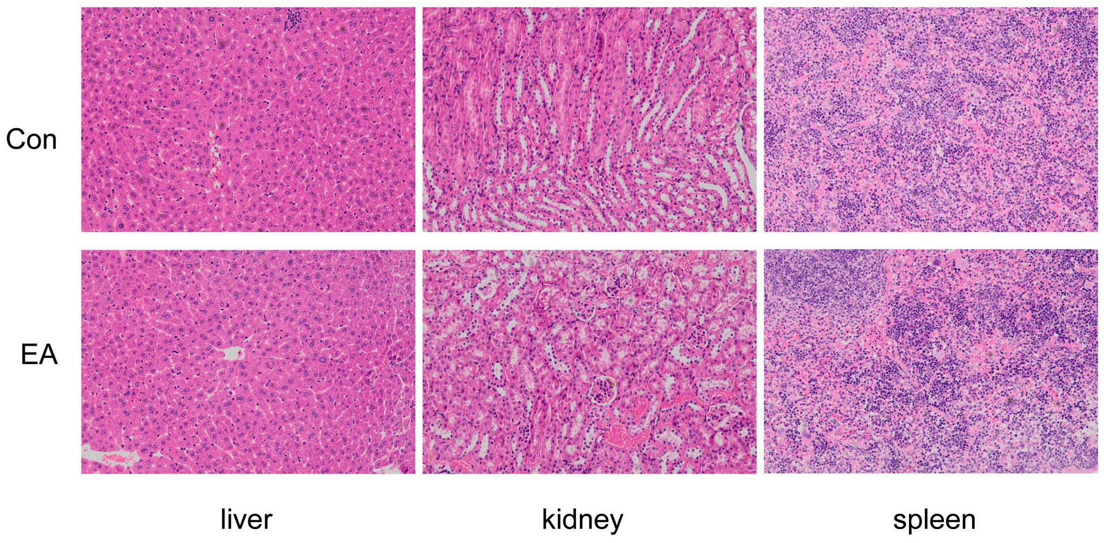

Figure S8. Pathological effects of high-dose EA treatment on the liver, kidney, and spleen of mice.
